# One year of high-precision operational data including measurement uncertainties from a large-scale solar thermal collector array with flat plate collectors, located in Graz, Austria

**DOI:** 10.1016/j.dib.2023.109224

**Published:** 2023-05-10

**Authors:** Daniel Tschopp, Philip Ohnewein, Roman Stelzer, Lukas Feierl, Marnoch Hamilton-Jones, Maria Moser, Christian Holter

**Affiliations:** a AEE — Institute for Sustainable Technologies (AEE INTEC)^2^, Feldgasse 19, 8200 Gleisdorf, Austria; b Forschung Burgenland, Campus Eisenstadt, Campus 1, 7000 Eisenstadt, Austria; c SOLID Solar Energy Systems GmbH (SOLID)^3^, Am Pfangberg 117, 8045 Graz, Austria; d Graz University of Technology^4^, Institute of Software Technology, Inffeldgasse 16b/II, 8010 Graz, Austria; e solar.nahwaerme.at Energiecontracting GmbH, Puchstrasse 85, 8020 Graz, Austria

**Keywords:** Renewable heat, Solar district heating, Large-scale solar thermal plant, In situ measurement, Radiation data, Quality assurance, Performance monitoring

## Abstract

This work presents operational data of a large-scale solar thermal collector array. The array belongs to a solar thermal plant located at Fernheizwerk Graz, Austria, which feeds into the local district heating network and is one of the largest Solar District Heating installations in Central Europe. The collector array deploys flat plate collectors with a total gross collector area of 516 m^2^ (361 kW nominal thermal power). Measurement data was collected in situ within the scientific research project MeQuSo using high-precision measurement equipment and implementing extensive data quality assurance measures. Data compromises one full operational year (2017) in a 1-minute sampling rate with a share of missing data of 8.2%. Several files are provided, including data files and Python scripts for data processing and plot generation. The main dataset contains the measured values of various sensors, including volume flow, inlet and outlet temperature of the collector array, outlet temperatures of single collector rows, global tilted and global horizontal irradiance, direct normal irradiance, and weather data (ambient air temperature, wind speed, ambient relative humidity) at the plant location. Beyond the measurement data, the dataset includes additional calculated data channels, such as thermal power output, mass flow, fluid properties, solar incidence angle and shadowing masks. The dataset also provides uncertainty information in terms of standard deviation of a normal distribution, based either on sensor specifications or on error propagation of the sensor uncertainties. Uncertainty information is provided for all continuous variables, with some exceptions such as the solar geometry, where uncertainty is negligible. The data files include a JSON file containing metadata (e.g., plant parameters, data channel descriptions, physical units, etc.) in both human and machine-readable format. The dataset is suitable for detailed performance and quality analysis and for modelling of flat plate collector arrays. Specifically, it can be helpful to improve and validate dynamic collector array models, radiation decomposition and transposition algorithms, short-term thermal power forecasting algorithms with machine learning techniques, performance indicators, in situ performance checks, dynamic optimization procedures such as parameter estimation or MPC control, uncertainty analyses of measurement setups, as well as testing and validation of open-source software code. The dataset is released under a CC BY-SA 4.0 license. To the best knowledge of the authors, there is no comparable dataset of a large-scale solar thermal collector array publicly available.

**Specifications Table**

**Table:** 

Subject	Renewable Energy, Sustainability and the Environment
Specific subject area	Solar thermal collector array measurements
Type of data	Text file (comma-separated values), JSON file, Python scripts
How the data were acquired	Measurement data was acquired within the research project MeQuSo [1]. High-precision measurement devices were installed in situ, e.g., electromagnetic flow sensor OPTIFLUX 4000 DN32 IFC 100, resistance thermometers Pt100 (EN 60751 F.01), Pyranometer Kipp&Zonen CMP 11, Pyranometer Kipp&Zonen SMP 21, Pyrheliometer Kipp&Zonen SHP1, mounted on a SOLSYS 2 two-axis sun tracker with active tracking. The density and heat capacity of the heat transfer fluid used in the collector loop were determined in a dedicated laboratory test. The instrumentation was regularly inspected on-site, and data was meticulously quality-checked both automatically and manually.
Data format	Raw, analyzed
Description of data collection	The measurement data covers one full operational year (2017) and was recorded with a data logger in 1-second sampling at the installation. Data was then transferred via internet connection to AEE INTEC, where data quality checks, data pre-processing and resampling to a 1-minute sampling rate were performed using the MATLAB® based ADA software developed at AEE INTEC [2]. Additional data channels were calculated using a Python script available with the dataset.
Data source location	Measurement data was obtained at the large-scale solar thermal plant Fernheizwerk Graz. Institutions: AEE – Institute for Sustainable Technologies, SOLID Solar Energy Systems GmbH, solar.nahwaerme.at Energiecontracting GmbH Location: Graz, Austria Latitude, Longitude: 47.047294 °N, 15.436366 °E
Data accessibility	https://zenodo.org/record/7741084 https://gitlab.com/sunpeek/zenodo-fhw-arconsouth-dataset-2017

## Value of the Data

• To the best knowledge of the authors, there is no comparable dataset of a solar thermal collector array publicly available in terms of high-precision measurement instrumentation, scientific data quality assurance, inclusion of (propagated) measurement uncertainty, fluid property laboratory testing, sampling rate and detailed plant documentation including information about external shadowing.
• The collector array is representative of typical large-scale solar thermal plant designs (flat plate collectors, widely used hydraulic arrangement). The dataset shows a real-scale application, and covers all seasons (includes data from one full operational year).
• Beneficiaries of the data are research institutes, the solar thermal industry (plant operators, plant designers, collector manufacturers), data scientists, and software developers who can use these data for detailed performance analysis and modelling of collector arrays. The data enables collaborative initiatives for open-source software development that rely on publicly available datasets for code testing, validation and demonstration.
• The solar thermal industry benefits from increased performance transparency for real-scale applications compared to laboratory tests, which promotes the technology to decision-makers in the energy sector and investors.
• More specifically, the data can be helpful to improve and validate radiation decomposition and transposition algorithms, control and short-term thermal power forecasting algorithms with machine learning techniques, performance indicators, in situ performance checks, parameter estimation procedures, and uncertainty analyses of measurement setups.

## 1 Objective

The objective to compile this dataset was the generation of high-precision and high-resolution measurement data of large-scale solar thermal collector arrays for scientific research purposes. The dataset was generated during the research project MeQuSo [1]. The MeQuSo project developed a proof of concept of a new in situ collector array test method called D-CAT (Dynamic Collector Array Test) applicable to a variety of typical large-scale solar thermal flat plate collector arrays. The data was additionally used by AEE INTEC within the project CollFieldEff^+^ for the development of collector array models [3] and the project 'Accompanying Research Project Large-scale Solar Thermal Plants' for plant benchmarking and optimization [4]. A major driving force for publishing this dataset was the reliance of open-source software projects on publicly available datasets; the authors of this article are contributing to the development of the open-source software SunPeek for performance monitoring of large-scale solar thermal plants [5].

## 2 Data Description

### 2.1 Collector Array

The presented data is from a large-scale solar thermal collector array, which is part of a large-scale solar thermal plant located at Fernheizwerk Graz, Austria. By definition, large-scale solar thermal plants are installations with more than 500 m^2^ collector area or 350 kW nominal thermal power [6]. The whole plant has a gross collector area of 8206 m^2^ (5744 kW nominal thermal power). It feeds into the local district heating network and is one of the largest Solar District Heating installations in Central Europe [7]. A unique feature of the plant is the deployment of ten different collector types from seven manufacturers on the same site, including flat plate, parabolic trough, and heat pipe collectors. Table 1 has key data of the Fernheizwerk Graz installation.

**Table 1:** Parameter of plant Fernheizwerk Graz.

Parameter	Value
Name	Fernheizwerk Graz
Location	Graz, Austria
Latitude, longitude	47.047294 °N, 15.436366 °E
Altitude	344 m
Total gross collector area	8206 m^2^
Nominal thermal power*	5744 kW
Application	Feed-in to the district heating network of the City of Graz
Plant designer	SOLID Solar Energy Systems GmbH
Plant operator, data owner	solar.nahwaerme.at Energiecontracting GmbH

⁎ conversion factor of 0.7 kW nominal thermal power per m^2^ collector area according to [10].

**Fig. 1:**
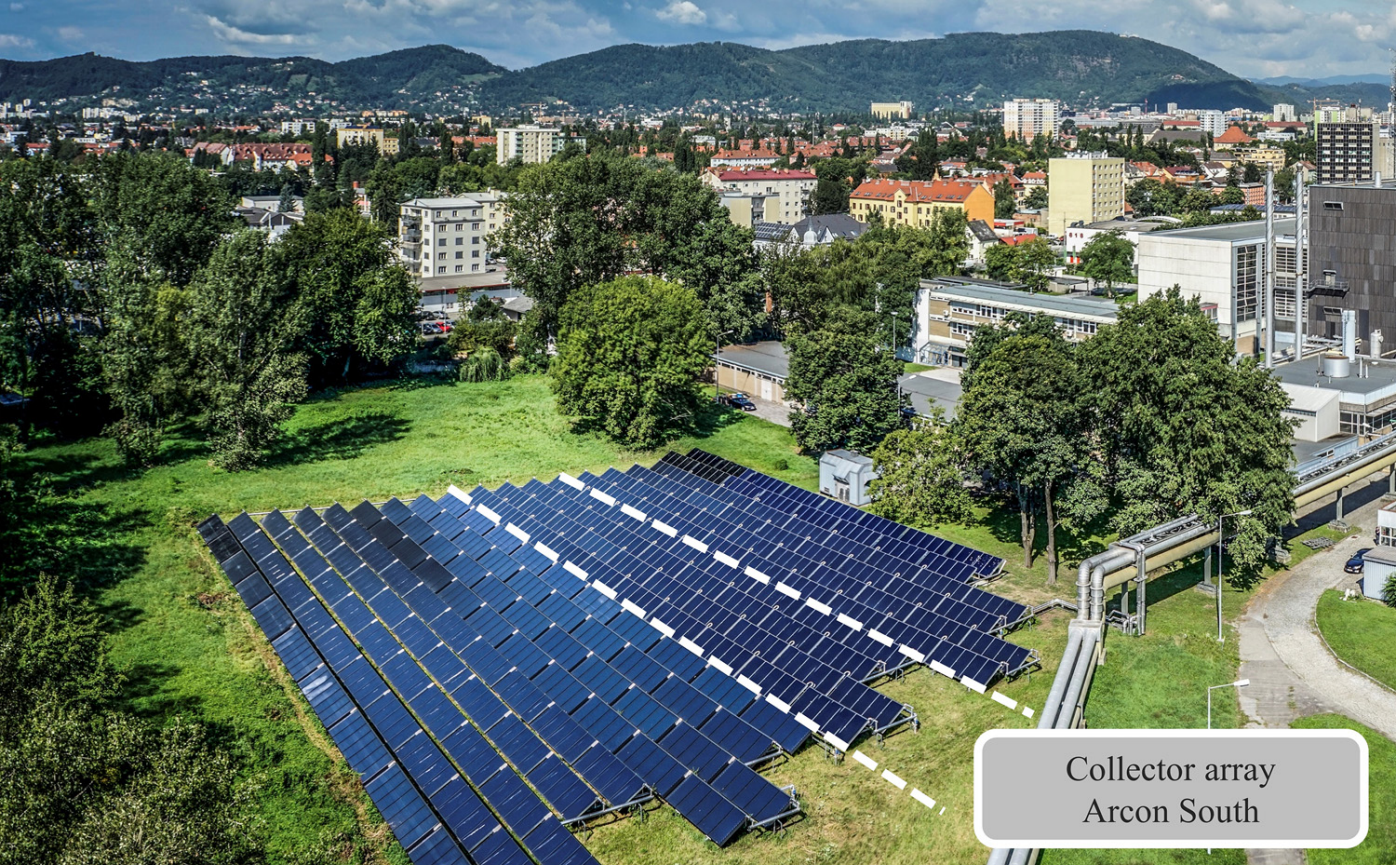
Collector array Arcon South (located between the white dashed lines) of plant Fernheizwerk Graz in 2017. View from the southeast. Source: Picfly.at Thomas Eberhard.

The data refers to the collector array Arcon South with flat plate collectors and a total gross collector area of 516 m^2^ (361 kW nominal thermal power), as depicted in Fig. 1. The collector array consists of four parallel collector rows with a common inlet and outlet connection. Collectors all face south direction (180°), have a tilt angle of 30°, and a row spacing of 3.1 m (see Table 2). The array deploys large-scale flat plate collectors of Arcon-Sunmark A/S (see Table 3). This collector type is very common for large-scale solar thermal plants, and the collector model 'HTHEATstore 35/10' is one of the most widely used, especially in Denmark, the world's leading market in Solar District Heating [8]. In 2020, Arcon-Sunmark A/S production lines were acquired by the company GREENoneTEC, who continue to produce a modified version of the collector under the brand name ‘GK HT 13,6’ [9].

**Table 2:** Parameters of collector array Arcon South to which the dataset refers.

Parameter	Value
Commissioning year	2014
Collector brand and model	Arcon-Sunmark A/S, 'HTHEATstore 35/10'
Mounting	Ground-mounted on grass field
Total gross collector area	515.66 m^2^
Nominal thermal power	360.96 kW
Orientation (azimuth)	180° (south)
Tilt	30°
Row spacing	3.1 m
Mounting level (distance between ground and collectors)	435 mm
Ground tilt	0° (horizontal)
Heat transfer fluid in collector loop	Propylene glycol (volume concentration: 43.5%)
Total fluid volume	0.4721 m^3^
Number of collectors	Row 1: 10, Row 2: 10, Row 3: 9, Row 4: 9, Total: 38

**Table 3:** Parameters of collector ‘HTHEATstore 35/10’ [11].

Parameter	Value
Collector type	Flat plat collector, double glazed with glass cover and foil
Absorber type	Harp, absorber stripes
Gross collector area	13.57 m^2^
Gross length	5973 mm
Gross width	2272 mm
Gross height	145 mm
n_0,b_	0.745
K_d_	0.930
n_0,hem_*	0.737
a_1_	2.067 W/(m^2^ K)
a_2_	0.009 W/(m^2^ K^2^)
a_3_	0.000 J/(m^3^ K)
a_4_	0.000
a_5_	7.313 kJ/(K m^2^)
a_6_	0.000 s/m
IAM	*Angles: 10°, 20°, 30°, 40°, 50°, 60°, 70°, 80°, 90°*;
	IAM longitudinal: 1.00, 0.99, 0.97, 0.94, 0.90, 0.82, 0.65, 0.32, 0.00; IAM transversal: 1.00, 0.99, 0.97, 0.94, 0.90, 0.82, 0.65, 0.32, 0.00

⁎ calculated according to ISO 9806:2017 [12]: n_0,hem__=_n_0,b_ (0.85 + 0.15 K_d_).

### 2.2 Available Data Channels

**Fig. 2:**
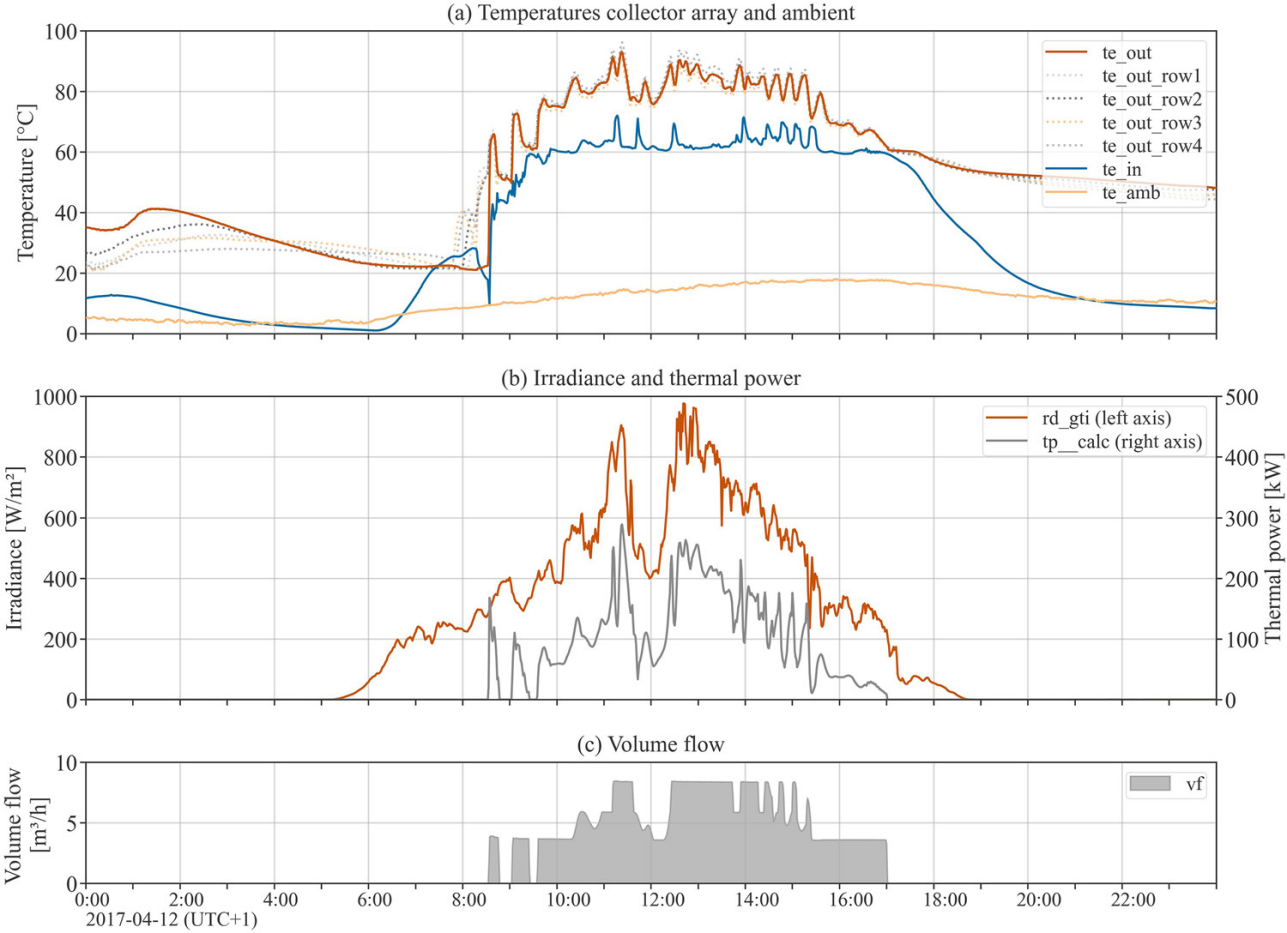
Array operation: Example day plot of selected data channels related to collector array operation (see Table 4) and global tilted irradiance (see Table 6). Note the outlet temperatures of each of the 4 collector rows in the top subplot.

**Fig. 3:**
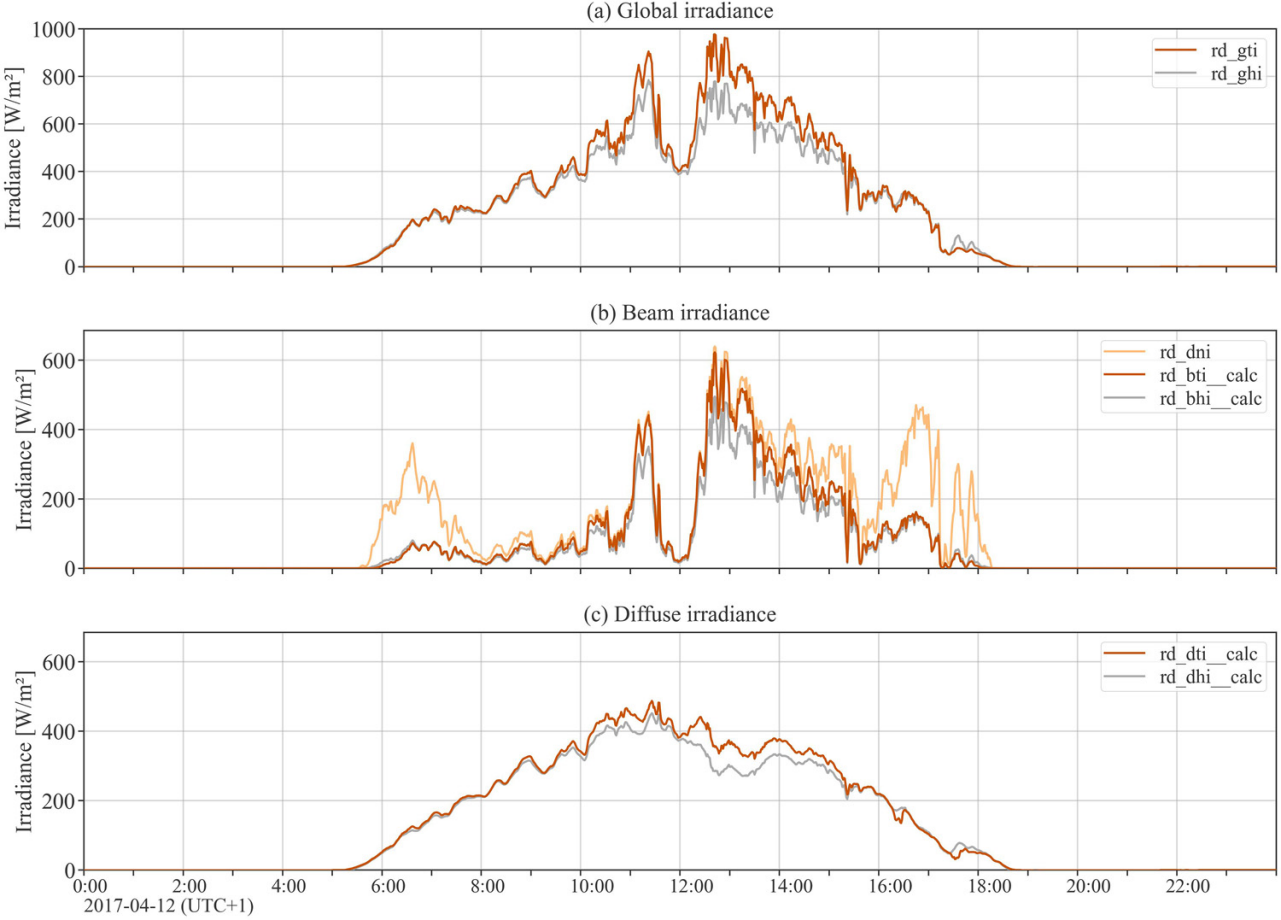
Irradiance: Example day plot of all irradiance-related data channels (subset of Table 6).

The dataset contains high-precision measurement data for one full operational year (2017) related to collector array Arcon South, with a sampling rate of 1-minute. Data include the measured values of volume flow, inlet and outlet temperature of the collector array, outlet temperatures of single collector rows, global tilted and global horizontal irradiance, direct normal irradiance, and weather data (ambient air temperature, wind speed, ambient relative humidity) at the plant location. Beyond the measurement data, the dataset includes additional calculated data channels that are helpful for performance analysis, such as thermal power output, mass flow, fluid properties, solar incidence angle and shadowing masks. Table 4, Table 5, Table 6, Table 7 hold complete data channel lists (without uncertainty information). Fig. 2, Fig. 3 show plots of selected data channels for an example day.

**Table 4:** Data channels related to collector array operation (uncertainty data channels omitted).

Data channel	Unit	Description
vf	m^3^/s	Total volume flow of collector array.
mf_calc	kg/s	Total mass flow of collector array.
tp__calc	W	Total thermal power output of collector array.
te_in	K	Inlet temperature to the collector array.
te_out	K	Outlet temperature from the collector array (all rows joined).
te_out_row1	K	Outlet temperature from collector array row 1.
te_out_row2	K	Outlet temperature from collector array row 2.
te_out_row3	K	Outlet temperature from collector array row 3.
te_out_row4	K	Outlet temperature from collector array row 4.

**Table 5:** Data channels related to fluid properties (uncertainty data channels omitted).

Data channel	Unit	Description
rho_in__calc	kg/m^3^	Density of heat transfer fluid at collector array inlet.
rho_out__calc	kg/m^3^	Density of heat transfer fluid at collector array outlet.
cp_in__calc	J/(kg K)	Heat capacity of heat transfer fluid at collector array inlet.
cp_out__calc	J/(kg K)	Heat capacity of heat transfer fluid at collector array outlet.

**Table 6:** Data channels related to weather (uncertainty data channels omitted).

Data channel	Unit	Description
rd_gti	W/m^2^	Global irradiance in plane of collector array.
rd_ghi	W/m^2^	Global horizontal irradiance.
rd_bti__calc	W/m^2^	Beam / direct irradiance in plane of collector array. Potential beam shadowing on the collectors is not taken into account.
rd_bhi__calc	W/m^2^	Beam / direct horizontal irradiance.
rd_dni	W/m^2^	Direct normal irradiance.
rd_dti__calc	W/m^2^	Diffuse irradiance in plane of collector array. Diffuse masking on the collectors due to front collector rows is not taken into account.
rd_dhi__calc	W/m^2^	Diffuse horizontal irradiance.
te_amb	K	Ambient air temperature.
ve_wind	m/s	Horizontal wind speed / wind velocity at the top edge of the collectors.
rh_amb	dimensionless	Ambient relative humidity.

**Table 7:** Data channels related to sun properties and shadowing.

Data channel	Unit	Description
aoi__calc	deg	Angle of incidence between the normal vector of the collector plane and the sun-beam vector.
sun_azimuth__calc	deg	Solar azimuth angle.
sun_apparent_elevation__calc	deg	Apparent solar elevation / altitude angle.
is_shadowed__calc	dimensionless	Binary variable. True if the collector array, at a particular timestamp, is considered shadowed (either partly or completely) by any shadow type.
is_shadowed_external	dimensionless	Binary variable. True if the collector array, at a particular timestamp, is considered shadowed (either partly or completely) by external objects.
rd_bti_shadowed_share__calc	dimensionless	Float between 0 (not shaded) and 1 (completely shaded). Degree of beam shadowing caused by front collector rows due to row-to-row shadowing.
is_shadowed_internal__calc	dimensionless	Binary variable. True if the collector array, at a particular timestamp, is considered shadowed (either partly or completely) by internal / row-to-row shadowing.

### 2.3 Shadowing

**Fig. 4:**
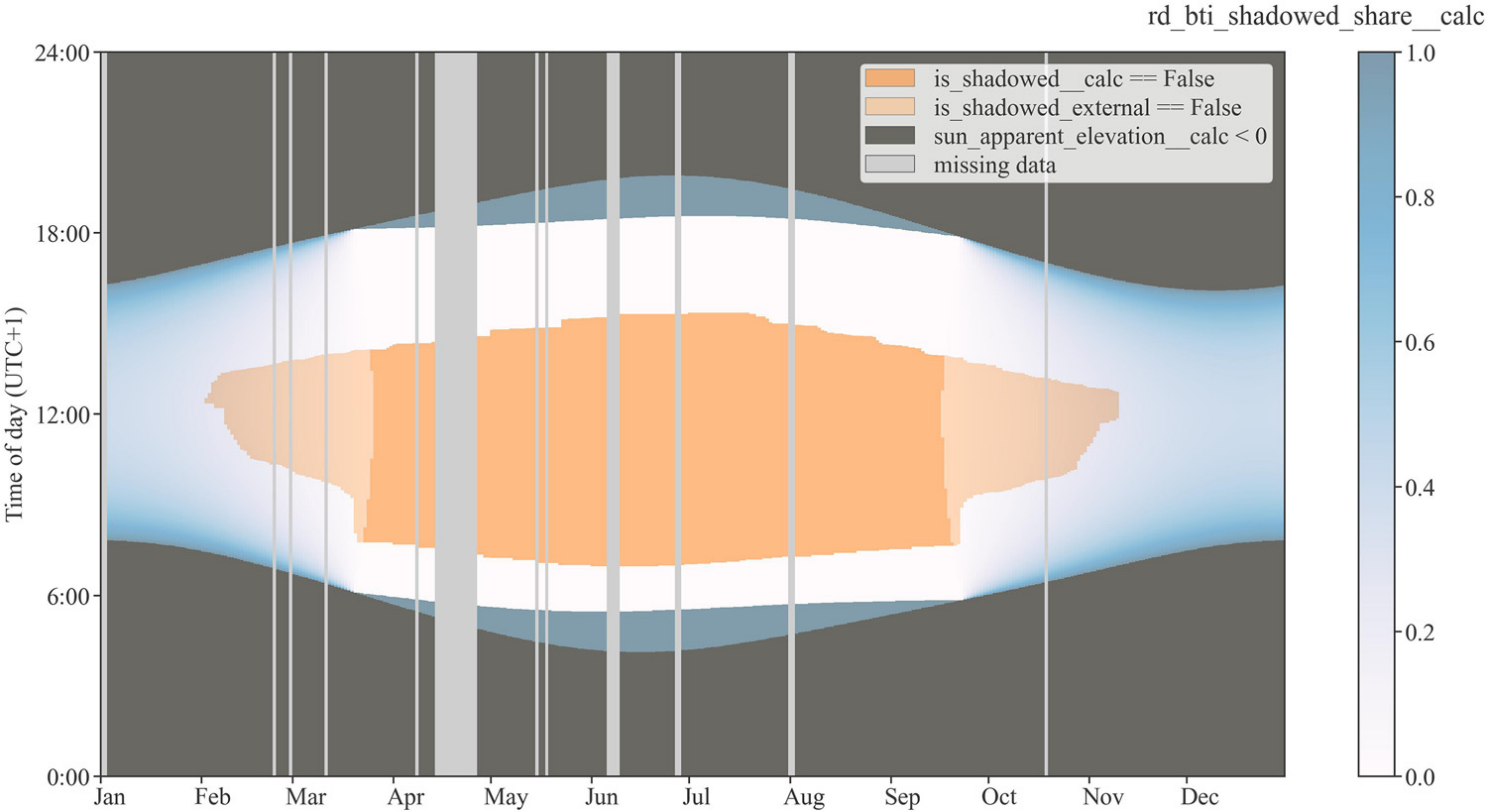
Array shadowing: Internal and external shadowing data channels (subset of Table 7) for the full measurement period. Periods with no shadowing are in orange, periods with no external shadowing in bright orange. Colour bar shows share of internal (row-to-row) beam shadowing. Vertical grey lines show missing data.

Some performance analysis methods like the power performance check according to ISO 24194:2022 [13] require filtering out operational periods where shadowing of any type affects the collector array. For the plant Fernheizwerk Graz, external shadowing is a major issue. As shown in Fig. 1, there are multiple shadowing objects in close vicinity. Towards east, the transport pipe of the district heating grid with a height of approx. 3 m is installed in close proximity to the collector array; towards south and west there are buildings and trees (at a distance of approx. 20 to 50 m). To precisely determine external shadowing, a 3D model of the array was set up as part of a master thesis at AEE INTEC [14]. For further details, see [1]. Fig. 4 shows the data channels referring to internal shadowing, external shadowing and the combination of both for the measurement period.

**Fig. 5:**
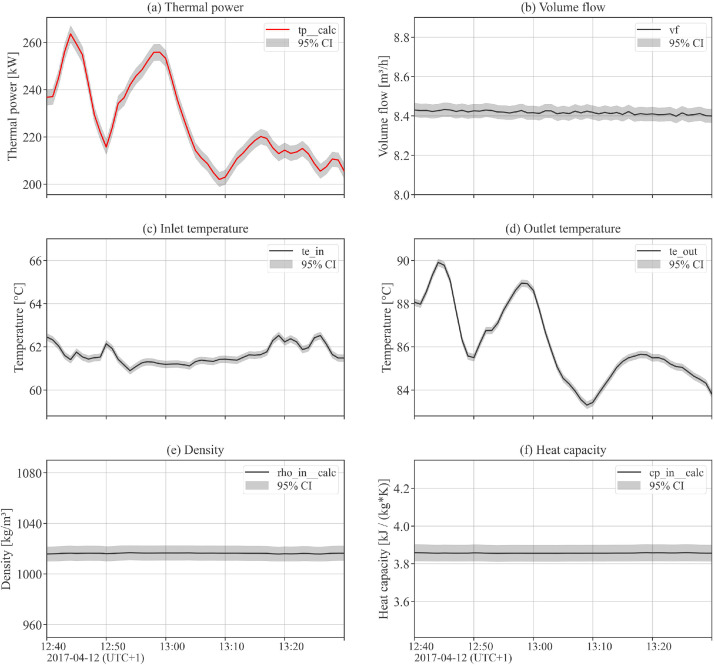
Example uncertainty plot with 95% confidence intervals, CI=±1.96u(y(t)).Subplot (a) shows the calculated values and confidence intervals for the thermal power output, based on Eq. (1) and error propagation on the input data channels in subplots (b) to (f).

### 2.4 Uncertainty Information

The dataset also provides uncertainty information for the measured and calculated data channels in the form of additional uncertainty data channels. Uncertainty information is given in terms of standard deviation of a normal distribution u(y(t)), based on sensor specifications or on error propagation of the measurement uncertainties (see Sections 3.2 and 3.3 for more details). Uncertainty information is available for all continuous variables with the exception of solar geometry based properties where uncertainty is negligible (*aoi__calc, sun_azimuth__calc, sun_apparent_elevation__calc, rd_bti_shadowed_share__calc*). Also, binary variables (*is_shadowed__calc, is_shadowed_external, is_shadowed_internal__calc*) have no measurement uncertainty assigned.

Fig. 5 shows an example uncertainty plot for thermal power output. The thermal power *tp__calc* is calculated based on measured data channels as

(1) tp__calc=vf·rho_in__calc·cp_in__calc·(te_in−te_out)

### 2.5 Data Gaps

**Fig. 6:**
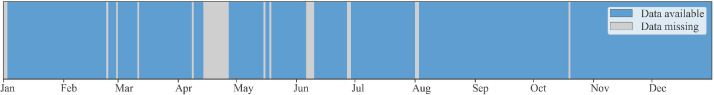
Data availability: Periods with available data are shown in blue, missing data periods in grey.

Fig. 6 and Table 8 provide an overview of the available data and missing data (data gaps). In the provided CSV files, missing data are encoded with no symbol (two subsequent separators). For background information on data gaps see Section 3.4. To ease the practical use, missing data are organized in blocks, meaning that data gaps affect the whole day and all channels. For a particular day, all data channels have either valid values for all timestamps or no data is available. Overall, data for 30 days is missing (8.2%), with one major gap in the month of April.

**Table 8:** Data gaps. All gaps are multiples of whole days.

Start	End	Number of days
2017–01–01 00:00	2017–01–02 23:59	2
2017–02–23 00:00	2017–02–23 23:59	1
2017–02–28 00:00	2017–02–28 23:59	1
2017–03–11 00:00	2017–03–11 23:59	1
2017–04–08 00:00	2017–04–08 23:59	1
2017–04–14 00:00	2017–04–26 23:59	13
2017–05–15 00:00	2017–05–15 23:59	1
2017–05–18 00:00	2017–05–18 23:59	1
2017–06–06 00:00	2017–06–09 23:59	4
2017–06–27 00:00	2017–06–28 23:59	2
2017–08–01 00:00	2017–08–02 23:59	2
2017–10–19 00:00	2017–10–19 23:59	1

### 2.6 Data Files

The following data files are provided:

• **FHW_ArcS__main__2017.csv** – This is the main dataset. It is advised to use this file for further analysis. The file contains the full time series of all measured and calculated data channels and their (propagated) measurement uncertainty. Calculated data channels are derived from measured channels (see script *make_data.py* below) and have the suffix *__calc* in their channel names. Uncertainty information, where available, is given in terms of standard deviation of a normal distribution (suffix *__std*).
• **FHW_ArcS__main__2017.parquet** – Same as *FHW_ArcS__main__2017.csv*, but in parquet file format for smaller file size and improved performance when loading the dataset in software.
• **FHW_ArcS__parameters.json** – Contains various metadata about the dataset, in both human and machine-readable format. Includes plant parameters, data channel descriptions, physical units, etc.
• **FHW_ArcS__raw__2017.csv** – Dataset with time series of all measured data channels and their measurement uncertainty. The main dataset *FHW_ArcS__main__2017.csv*, which includes all calculated data channels, is a superset of this file.

Additionally, the following Python scripts are provided:

• **make_data.py** – This Python script exposes the calculation process of the calculated data channels (suffix *__calc*), including error propagation. The main calculations are defined as functions in the module *utils_data.py*.
• **make_plots.py** – This Python script, together with *utils_plots.py*, generates several figures displayed in this paper, based on the main dataset.

## 3 Experimental Design, Materials and Methods

### 3.1 Measurement Setup

**Fig. 7:**
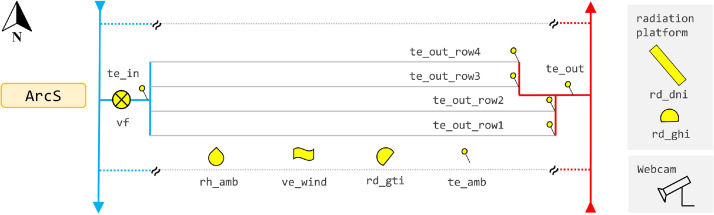
Measurement setup.

Measurement data was acquired within the research project MeQuSo, where the solar thermal plant Fernheizwerk Graz was equipped with high-precision measurement equipment in mid-2016 [1]. Fig. 7 shows the measurement setup, Table 9 provides the sensor specifications and information on the calibration procedure. Sensor calibration took place in mid-2016.

**Table 9:** Sensor specifications.

Name	Measured quantity	Sensor	Calibration
vf	Volume flow	Electromagnetic flow sensor OPTIFLUX 4000 DN32 IFC 100	In situ at plant Fernheizwerk Graz
te_in, te_out, te_out_row1–4	Fluid temperature	Resistance Thermometer Pt100 (EN 60751 F.01), without thermowell	Laboratory AEE INTEC
te_amb	Ambient air temperature	Resistance Thermometer Pt100 (EN 60751 F.01), with ventilation unit	manufacturer
rd_ghi	Global horizontal irradiance	Pyranometer Kipp&Zonen CMP 11	In situ at plant Fernheizwerk Graz
rd_gti	Global tilted irradiance	Pyranometer Kipp&Zonen SMP 21	manufacturer
rd_dni	Direct normal irradiance (DNI)	Pyrheliometer Kipp&Zonen SHP1, mounted on SOLSYS 2 sun tracker with active tracking	manufacturer
ve_wind	Wind speed horizontal	Ultrasonic Wind Sensor Lufft V200A-UMB	manufacturer
rh_amb	Ambient relative humidity	Epulse EE071 HCT01–00D	manufacturer
Data logger	PLC B&R Industrial Automation X20CP1483, input modules B&R X20AI4222 for vf and rd_ghi (4–20 mA analogue signal, 12 bit A/D resolution, 100 ms sampling), input modules B&R X20ATB312 for te_in, te_out, te_out_row1–4 (four wire connection, 24 bit A/D resolution, 100 ms sampling), input modules B&R X20AI4222 for te_amb and rh_amb (0–10 V analogue signal, 12 bit A/D resolution, 100 ms sampling), Modbus-RTU fieldbus communication for rd_gti, rd_dni and ve_wind.		

To meet the installation requirements of the volume flow sensor regarding minimum inflow and outflow pipe lengths, the manifold pipe leading to the four collector rows was extended to include a flow-calming section. The inlet and outlet temperatures of the array are measured in the connection pipes right before and after the collector rows. All fluid temperature sensors are placed in counter-flow direction and are directly immersed in the fluid (without thermowell), in order to reduce response time. Fluid temperature sensors have a four wire (4 L) connection to the data logger to compensate for the lead wire resistance.

Global tilted irradiance, wind speed, ambient air temperature and relative humidity are measured in a neighbouring collector array, about 3 m from the first collector row. The pyranometer to measure the global tilted irradiance is placed on top of the collector, which implies that the recorded values are higher than the beam and diffuse irradiance average over the array, due to shadowing and masking effects [15]. The sensors to measure direct normal irradiance and global horizontal irradiance are placed on a platform, about 50 m east of the location of the global tilted irradiance sensor. In order to avoid view obstructions, the platform is mounted 3 m above the ground, looming over the district heating transport line. To the southeast, a webcam was installed for visual impression on shadowing effects and vegetation growth, to detect major faults, and for documentation of additional relevant events. The total cost of the measurement equipment was in the range of 20 – 30 k€.

### 3.2 Measurement Uncertainty of Sensors and Fluid Properties

Sensors were calibrated and installed in mid-2016, about half a year before the data collection for the presented dataset started. The last column of Table 9 lists the calibration method. All fluid temperature sensors were calibrated in the laboratory of AEE INTEC at 60 °C and 87 °C. The volume flow sensor was calibrated in the field with a high-precision reference sensor for 5 points (15%, 40%, 60%, 80%, and 100% of the nominal volume flow). Pyranometer CMP 11 was calibrated on the radiation platform in reference to pyranometer SMP 21, which was mounted temporarily on the sun tracker, and pyrheliometer SHP 1. For further details, see [1].

All measured data channels and calculated fluid properties provided with this dataset include a native measurement uncertainty using information in Table 10, Table 11, Table 12. Measurement uncertainties of the deployed sensors were determined based on data sheet specifications. For a particular sensor, multiple uncertainty sources may exist, such as zero off-set, non-stability, non-linearity etc. for radiation sensors (see Table 11). Uncertainty sources were combined into a total sensor uncertainty, expressed as standard deviation of a normal distribution according to GUM [16]. In applying this procedure, each measured value y(t) is assigned a corresponding standard deviation u(y(t)) of a normally distributed error unc_dist(y(t)).

(2) y(t)↦u(y(t));unc_dist(y(t))∼N(y(t),u(y(t))

**Table 10:** Uncertainty sources of installed sensors (without radiation sensors).

Name	Uncertainty source	Distribution	Value
vf	General	Uniform	±0.3% of MV, additional ±1 mm/s for DN32 (= 8.3 * 10^−7^ m^3^/s)
	Repeatability	Uniform	±0.1% of MV, minimum ± 1 mm/s for DN32 (= 8.3 * 10^−7^ m^3^/s)
	Non-stability	Uniform	±0.1% of MV
te_in, te_out, te_out_row1–4, te_amb	General	Uniform	±0.1 *K* + min(|0.00167 * (MV – te_cal)|)
			te_cal (fluid temperatures) = [60 °C, 87 °C]
			te_cal (ambient air temperature) = 0 °C
	Non-stability	Uniform	±0.1 K / year (used value: 1 year)
ve_wind	General	Normal (σ)	±3% of MV, minimum 0.173 m/s
rh_amb	General	Uniform	±2% of MV for values in [0%,90%) ±3% of MV for values in [90%, 100%]

MV… measured value, te_cal… calibration temperatures.

**Table 11:** Uncertainty sources of radiation sensors.

	rd_ghi	rd_gti	rd_dni
Zero offset	±9 W/m^2^	±2 W/m^2^	±1 W/m^2^
Non-stability	±0.5% of MV	±0.5% of MV	±0.5% of MV
Non-linearity	±0.2% of MV	±0.2% of MV	±0.2% of MV
Directional response	±10 W/m^2^	±10 W/m^2^	–
Temperature response	±1% of MV	±0.3% of MV	±0.5% of MV
Spectral response	±1% of MV	±1% of MV	–
Tilt response	±0.2% of MV	±0.2% of MV	–

MV… measured value, all errors are normally distributed.

**Table 12:** Uncertainty sources of fluid properties [17].

Name	Uncertainty Source	Distribution	Value
rho_in__calc, rho_out__calc	General	Uniform	±0.5% of MV
cp_in__calc, cp_out__calc	General	Uniform	±1.0% of MV

MV… measured value.

To determine the density and heat capacity of the fluid in the collector loop (propylene glycol at a volume concentration 43.5%), a laboratory test was conducted at ILK Dresden [17]. Density was determined in 20 K steps and heat capacity in 10 K steps over the temperature range 20 °C to 120 °C. The density and heat capacity laboratory measurement values are listed in the metadata file *FHW_ArcS__parameters.json*. The fluid property uncertainties, as reported by ILK Dresden, are listed in Table 12. For details about these fluid property calculations, see *calc_fluid_prop()* in the Python file *utils_data.py*.

Binary variables have no measurement uncertainty assigned. Solar geometry based properties (*aoi__calc, sun_azimuth__calc, sun_apparent_elevation__calc, rd_bti_shadowed_share__calc*) are calculated based on the Python *pvlib* package [18] and have negligible uncertainty. The uncertainty of the data logger is not included in the sensor uncertainties. If data logger uncertainties were to be added by users of this dataset, it is recommended to set them in the range of ±0.10% - 0.15% of the measured value (uniform distribution), as these values have been used for similar setups [19].

### 3.3 Error Propagation

Calculated data channels in the main dataset have a suffix *__calc* (e.g., *tp__calc*) attached to their name and their corresponding uncertainty a suffix *__calc__std* (e.g., *tp__calc__std*). The uncertainty of calculated data channels is derived using GUM error propagation [16]. The standard uncertainty u(y) of the calculation output $Y=f(X_{1},X_{2},\;\ldots ,\;X_{2})$ using inputs $X\;=(X_{1},X_{2},\;\ldots ,\;X_{N})\;$can be approximated with

(3) $$u\left(y\right)=\sqrt{\sum_{i=1}^{N}{\left(\frac{\partial f}{\partial X_{i}}\right)}^{2}u{\left(x_{i}\right)}^{2}}$$

All calculations Y=f(X) in the provided dataset are linear functions of X. Hence, the Taylor series approximation in Eq. (3) is exact and the measurement uncertainties can be propagated without information loss using Eq. (3).

In terms of Python implementation, the error propagation for the calculated data channels is implemented using the *uncertainties* package [20]. Values and their standard deviation are expressed as an *unumpy* array, behaving numerically much the same way a vanilla *numpy* array does. Calculations using *unumpy* arrays yield *unumpy* arrays, hence the implemented error propagation is completely automated. After all calculations are finished, measurement value and their standard deviation are represented as two different columns in a *pandas* DataFrame, included in the main dataset.

### 3.4 Data Quality Checks and Pre-Processing

To ensure high-quality measurement data, the following on-going quality assurance measures were performed amongst others (for details see [1]):

• Regular on-site inspection of the measurement equipment (typically every two weeks).
• Regular cleaning of radiation sensors (typically once a week).
• Regular inspection of the plant on-site as well as remote with webcam pictures and the plant visualization (typically once a month).
• Automated plausibility checks for physically implausible values during data import.
• Documentation of all plant events (e.g., maintenance work, power supply interruption).

**Fig. 8:**
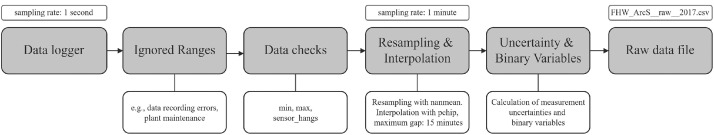
Data processing workflow.

The data logger recorded the data with 1-second sampling. Fig. 8 shows the applied pre-processing steps and quality checks, which were performed with the closed source MATLAB® based ADA software of AEE INTEC [2]. Ignored ranges are periods where data recording and transmission errors occurred, measurement instrumentation was maintained or the plant did not operate in the usual mode. Such events included installation of new measurement equipment for other collector arrays, power supply interruptions, cleaning of radiation sensors, grass cutting, insulation work, etc. These events occurred relatively often as the plant was part of a research project and construction work took place at the site. If an event occurred, the whole day was discarded for all data channels.

For days not discarded by defined ignored ranges, data checks were applied to the data, namely comparing against a lower and an upper threshold, and *sensor_hangs*, marking values that remain constant over a defined time period when they should actually vary (see Table 13). After the data checks, data was resampled to 1-minute sampling rate using *nanmean*. Data gaps remained at 70 intervals with a maximum length of 9 minutes; these were interpolated using *pchip* interpolation in MATLAB®. These processing steps did not lead to additional data gaps. Uncertainties of measured data channels were calculated on the resampled 1-minute values, assuming that resampling itself did not affect measurement uncertainty. Calculated data channels and their uncertainties (see Section 3.3) were calculated based on resampled data. Also, the binary variable *is_shadowed_external* (see Section 2.3) was created on the 1-minute time grid.

**Table 13:** Automated data checks.

Name	min	max	sensor_hangs
vf	−50 litre/h	–	–
te_in, te_out, te_out_row1–4	−30 °C	200 °C	24 h
te_amb	−30 °C	45 °C	3 h
rd_gti, rd_ghi, rd_dni	−10 W/m^2^	1500 W/m^2^	24 h
ve_wind	0 m/s	50 m/s	1 h
rh_amb	0%	100%	3 h

The main reason to provide resampled data in connection to this article was to substantially reduce the file size and minimize distortions resulting from the sensor response times. For an overview of available data and missing data (data gaps) see Section 2.5, Fig. 6 and Table 8.

## Ethics Statements

No ethical issues are associated with this work.

## CRediT authorship contribution statement

**Daniel Tschopp:** Conceptualization, Methodology, Software, Validation, Data curation, Writing – original draft, Writing – review & editing, Visualization. **Philip Ohnewein:** Conceptualization, Methodology, Software, Validation, Data curation, Writing – review & editing, Visualization, Supervision. **Roman Stelzer:** Conceptualization, Methodology, Software, Data curation. **Lukas Feierl:** Methodology, Software, Visualization. **Marnoch Hamilton-Jones:** Methodology, Software, Data curation. **Maria Moser:** Project administration, Funding acquisition. **Christian Holter:** Resources, Supervision, Project administration, Funding acquisition.

## Declaration of Competing Interest

The authors declare the following financial interests/personal relationships which may be considered as potential competing interests: Lukas Feierl, Maria Moser and Christian Holter are employed at SOLID Solar Energy Systems GmbH, Christian Holter is employed at solar.nahwaerme.at Energiecontracting GmbH.

## Data Availability

One year of high-precision operational data including measurement uncertainties from a large-scale solar thermal collector array with flat plate collectors, located in Graz, Austria (Original data) (Zenodo).
